# Flipped Classroom (FCR) as an Effective Teaching-Learning Module for a Large Classroom: A Mixed-Method Approach

**DOI:** 10.7759/cureus.28173

**Published:** 2022-08-19

**Authors:** Manpreet Kaur, Soumen Manna, Himani Ahluwalia, Manasi Bhattacharjee

**Affiliations:** 1 Physiology, Vardhman Mahavir Medical College and Safdarjung Hospital, New Delhi, IND; 2 Physiology, All India Institute of Medical Sciences, Guwahati, IND

**Keywords:** flipped classroom, teaching-learning, medical education, undergraduate and graduate medical education, teaching learning method

## Abstract

Flipped classroom (FCR) is one of the emerging active teaching-learning methods in the medical profession. Its potential for achieving learning objectives, especially in the scenario of a large classroom, especially in medical schools, has not been convincingly demonstrated. This study was designed to establish FCR model conduction and its overall utility as a teaching-learning methodology for undergraduate medical students in large classroom settings using a mixed-method approach using quantitative (assessment scores) and qualitative criteria (subjective feedback from students and teachers).

FCR was conducted for a batch of 170 first-year medical students for a hematology topic. Pre- and post-assessments (based on all the cognitive learning domains) were done to quantify the objective improvement after exposure to the FCR. In addition, subjective feedback from both students and teachers was taken on a validated feedback survey to decipher the qualitative benefits of the FCR.

Comparing pre- and post-assessment scores, there was a significant improvement after the FCR session, especially in the low performers. There was optimistic feedback from students and teachers regarding the utility of FCR as a teaching-learning module.

FCR as a teaching-learning module was feasible and effective, and the users seemed primarily satisfied. Although there is a higher workload for students and teachers, still FCR is an effective teaching-learning module for a large classroom.

## Introduction

Competency-based medical education (CBME) curriculum for undergraduate students emphasizes active learning strategies for teaching. Flipped classroom (FCR) approach is one of the promising active teaching-learning methodologies (TLM) and is gaining popularity in undergraduate medical education [1]. Therefore, there is an immense need to embrace the FCR model in medical education to develop highly vital application and analytical skills in our students [2].

FCR is a pedagogical approach that reverses or "flips" around the core elements of traditional didactic teaching [3,4]. It consists of distributing learning material to the learner before class, while what is traditionally termed homework is done in class [5]. Traditional lectures are primarily teacher-centered with a passive role for students [6]. On the other hand, FCR provides exposure to the study material a priori and stimulates students to prepare and participate in class activities [7]. The classroom becomes a student-centric learning space wherein the teacher can engage the students in various active learning strategies and address specific concepts that may need further clarification [8]. Thus, the role of the teacher is simply a facilitator/guide/mentor/advisor [9].

Present-day health profession educators aim to take learners’ skills to the higher levels of Bloom’s taxonomy. This requires the development of deeper cognitive processes such as critical thinking and analytical and evaluative skills. However, the traditional didactic lecture format of teaching-learning addresses only the lower levels of the cognitive domain; FCR has the potential to achieve higher levels of cognitive learning as well [10]. FCR is also known to promote the attitude toward lifelong learning, which is one of the desired attributes of a competent medical graduate [11]. Finally, FCR has an intrinsic ability to stimulate interaction and collaboration among learners [12].

Two significant challenges of FCR are the creation of effective content or pre-class material for viewing before class and optimum use of class time. The first challenge can be met at the level of the teacher by following the instructional design models for the preparation of pre-class learning material. Whereas the second challenge is a major issue, especially in cases of large group teaching, as it requires face-to-face interaction between students and teachers. This has been a predominant reason for its limited adoption in medical colleges, with student strength ranging beyond 100. Also, a limited number of faculty and facilitators poses an additional burden for the smooth conduction of in-class activity.

Large class size is a major hindrance to active learning strategies and the maintenance of student discipline. This is the most probable reason for the infrequent practice of FCR in large classroom settings despite its apparent advantages. Teachers assume that FCR conduction is not feasible in such huge class strengths as ensuring active team-based learning with the involvement of all students in this setting itself is a herculean task, besides the higher workload of planning and implementation of this module (more so in the case where technological support, teaching staff, and other educational resources are scarce).

Very few studies have used the FCR approach in large class sizes [13-18]. To establish any TLM, there are a few considerations, i.e., its feasibility, validity, effectiveness, and user satisfaction. FCR has been established as a valid active TLM. Its feasibility for small class sizes is beyond doubt, but studies are also trying to replicate this model for large groups by categorizing them into smaller teams. Compared to traditional lectures, the effectiveness of FCR has yielded conflicting results, with few reporting more [15,18], equal [13,17], or even less beneficial [14] in terms of scores achieved. Only one of the studies conducted in a large group has compared the pre- and post-test scores after exposure to FCR; this is important because the difference in scores between traditional lecture and FCR could be confounded easily by the basal knowledge of students about a particular subject [16]. Aristotle et al. also used a multiple-choice question (MCQ) pre- and post-assessment to gauge the effectiveness of FCR. MCQ-based assessment has some inherent limitations in itself that it tends to focus on the lower-level learning objectives (while FCR has more utility for developing higher-order thinking skills) and using them one cannot assess the students' abilities to organize, analyze, and synthesize ideas [16].

Moreover, the users for any TLM include both students and teachers. However, most studies on large group FCR have taken student feedback, and only one study has mentioned the perspective of a single teacher regarding FCR conduction [17]. The present study was planned to propose a feasible structure for conduction of FCR in a large classroom and establish its overall utility as TLM for undergraduate medical students in large class size settings with quantitative and qualitative criteria.

## Materials and methods

A cross-sectional interventional study was conducted on 170 first-year Bachelor of Medicine and Bachelor of Surgery (MBBS) undergraduate students at the Department of Physiology, Vardhman Mahavir Medical College and Safdarjung Hospital, New Delhi, India. The study was started after ethical approval (IEC/VMMC/SJH/ Project/ 2021-03/CC-138) with written informed consent from all the participants. An appropriate topic for the conduction of FCR was chosen by consensus of three subject experts. In addition, specific learning objectives were formulated for the selected topic based on the prescribed CBME competencies for the concerned topic in physiology (covering all the domains of cognitive learning).

Study design

**Figure 1:**
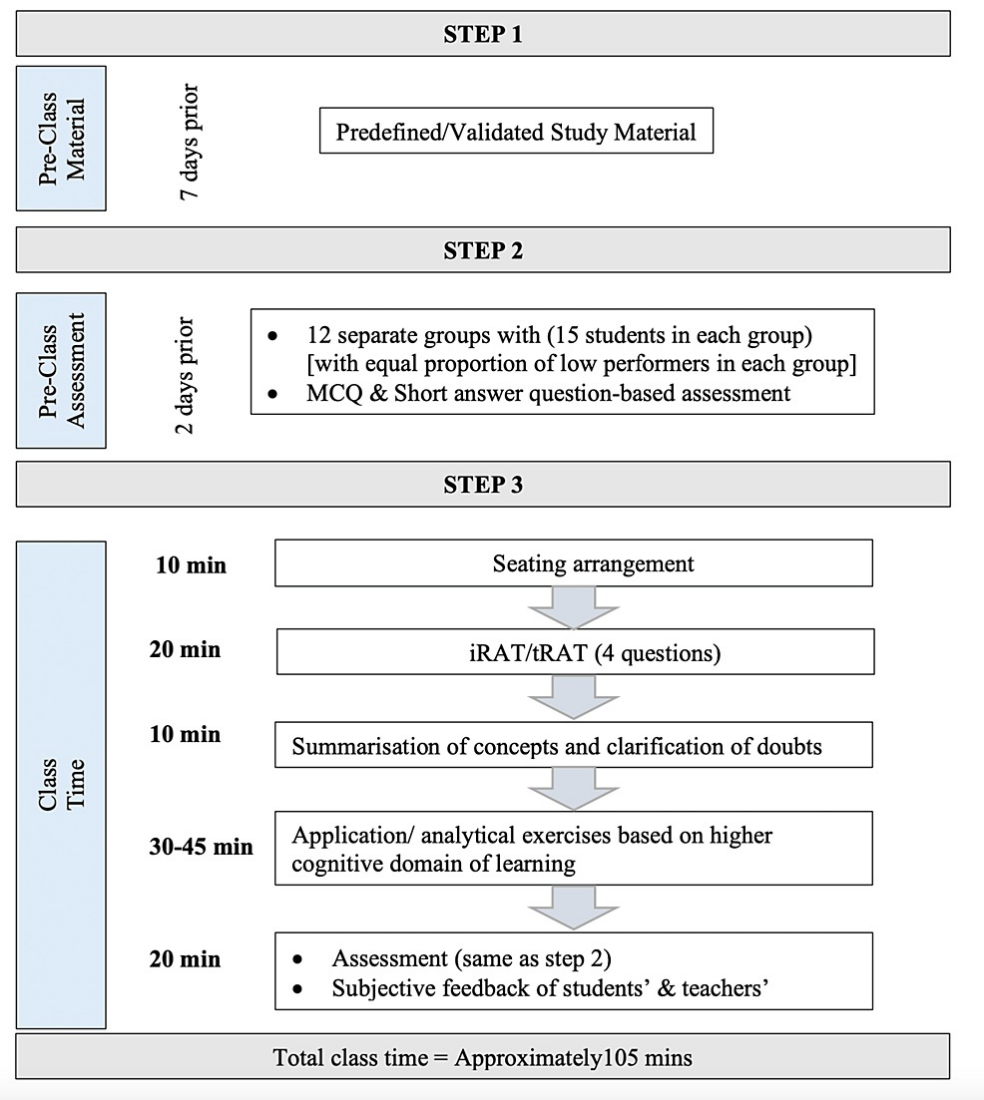
Study design MCQ: multiple-choice question; iRAT: individual readiness assurance test; tRAT: team readiness assurance test.

The study design is given in Figure 1.

Step 1

Seven days before the scheduled lecture, two short videos of 10 minutes duration with study materials were provided to the students using technology-enabled teaching tools. The study materials were created using instructional design models based on various theories of learning.

Step 2

Two days before the scheduled lecture, a pre-class assessment was done using Google Forms (Google LLC, Mountain View, CA), which included MCQs and short answer questions based on the pre-class material. Questions of the said assessment were designed to address all the specific learning objectives based on all cognitive learning domains. This assessment helped the teacher to decipher the strengths and weaknesses and thus decide on the concepts that would require more emphasis/clarification during class time. Also, using this assessment, the students were grouped using 50 percentile cut-off as low and high performers, and 12 separate groups with 15 students each with an equal proportion of low performers were made. Students were informed about these groups before the scheduled class and instructed to sit according to the groups assigned during class time.

Step 3

During the initial 10 minutes of class time, the students sat according to their assigned groups. The individual readiness assurance test (iRAT) was assessed using four MCQs in Google Forms. Then the same MCQs were given to groups, and the students were asked to solve them as a team to measure the team readiness assurance test (tRAT). After this, summarization of key concepts and clarification of students' doubts were done on the basis of pre-class assessment as well as discussion of any student queries regarding the topic. This was followed by assigning the application/analytical exercises formulated on the higher domains of cognitive learning to each group and their discussion. At the end of the session, post-assessment (same as pre-class assessment) objective scoring was done as well as the subjective feedback of students and teachers was also taken. The content of the entire FCR educational module was validated by three subject experts.

Subjective feedback was voluntary and anonymous. The questionnaire was first reviewed by the faculty members; they checked for the relevance and appropriateness of the items and any practical difficulties. This was done to validate the survey questions. Internal consistency was also calculated using Cronbach's alpha test, and the reliability coefficient for all the 10 items of the feedback questionnaire was found to be 0.87. This suggests that the items of the subjective feedback questionnaire are consistent and highly reliable.

Statistical analysis

After normality tests analysis, the data representation to Gaussian or non-Gaussian was deciphered, and on the basis of that, the data were either expressed as mean and standard deviation (if Gaussian distribution), otherwise as median and inter-quartile range (non-Gaussian distribution). The level of statistical significance was set at p < 0.05 (two-tailed). A comparison of pre- and post-assessment scores was done using the Mann-Whitney test. Also, the Cohen's D effect size for change in assessment scores was calculated. Finally, a comparison of the change in score based on quartiles made on their pre-FCR performance was made using the chi-square test. All statistical analyses were performed using GraphPad Prism version 5.00 for Windows (GraphPad Software, San Diego, CA).

## Results

The results of the study have been divided into three subheadings: readiness assurance test, assessment, and subjective feedback.

Part I: readiness assurance tests (individual and team)

Individual readiness assurance was assessed based on the individual responses of students to MCQs. The class responded fairly well individually, with the correct responses ranging from 70% to 92% (Table 1). However, when the teams discussed and attempted the same questions again, their performance improved but was not statistically significant. The range of improvement went to 99%, which suggests the significance of team-based learning methods in adult learning (Table 1).

**Table 1:** Scores in readiness assurance test iRAT: individual readiness assurance test; tRAT: team readiness assurance test.

Question number	Correct response rate in percentage individually - iRAT	Correct response rate in percentage after discussing as a team - tRAT
1	92%	94%
2	70%	83%
3	75%	88%
4	89%	99%

Part II: assessment

Pre-flipped Classroom Session (Pre-FCR) Assessment Scores

A total of 123 students had submitted the pre-FCR assessment, and the median score of the class was 12 out of 22, which is around 54.5% (Table 2).

**Table 2:** Average scores of students in assessments done before (pre-FCR) and after (post-FCR) flipped classroom FCR: flipped classroom.

	Pre-FCR score	Post-FCR score
Number of students	123	159
25% percentile	9.500	13.50
Median	12.00	16.00
75% percentile	14.50	17.00

Post-flipped Classroom Session (Post-FCR) Assessment Scores

A total of 159 students participated in class time sessions and completed the post-FCR assessment. Median scores of the students increased to 16 out of 22, which is about 72.7%, indicating a drastic improvement of around 18% in class performance (Table 2).

Comparison of Pre- and Post-FCR Assessment Scores

**Figure 2:**
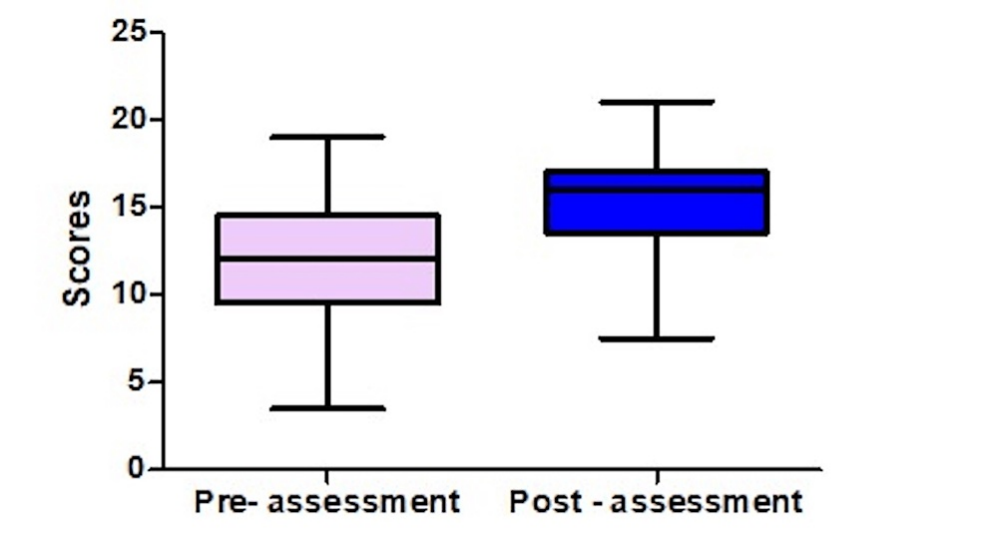
Comparison of pre- and post-assessment scores after exposure to a flipped classroom session

A total of 113 students had completed both the pre- and post-FCR assessment. We compared their scores and found a significant increase in their median score from 12 to 16 (Table 3 and Figure 2). We also calculated the Cohen's D effect size, which was equal to 1.05, indicating a large effect size with a significant impact.

**Table 3:** Comparison of pre- and post-FCR assessment scores in students who had attempted both assessments Mann-Whitney test with two-tailed p-value < 0.0001. FCR: flipped classroom.

	Pre-FCR	Post-FCR
Number of values	113	113
25% percentile	9.500	13.50
Median	12.00	16.00
75% percentile	14.50	17.00

**Figure 3:**
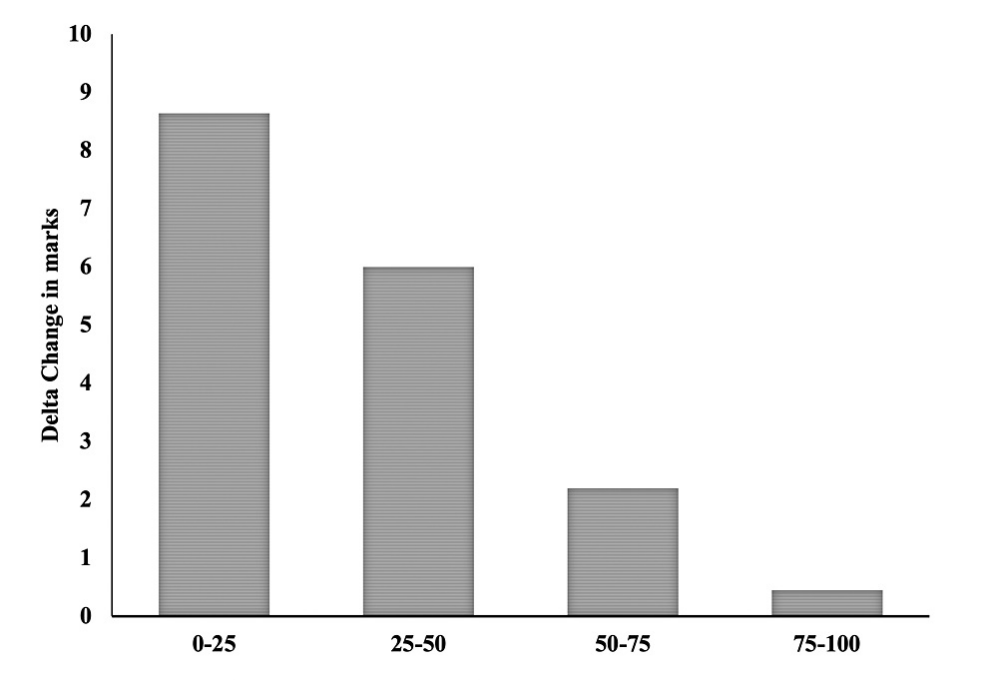
Comparison of change in scores based on pre-flipped classroom assessment score quartiles

We also assessed the change in performance (in terms of delta change in assessment score) after FCR, on the basis of their initial performance quartiles and found that 0-25% group (low performers: delta change = 8.64) > 25-50% group (delta change = 6) > 50-75% group (delta change = 2.2) > 75-100% group (high performers: delta change = 0.45) (Figure 3).

**Figure 4:**
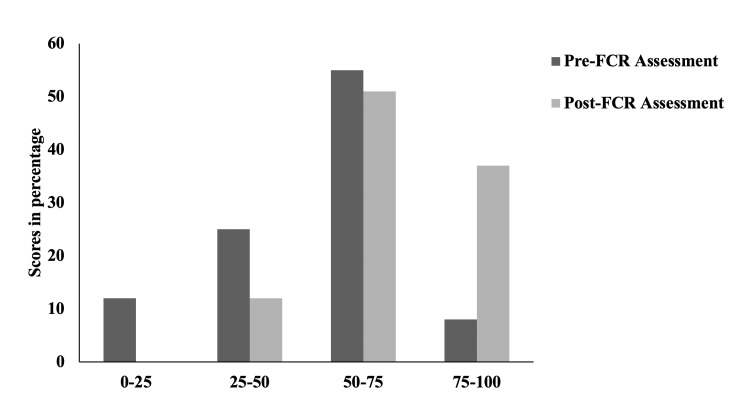
Comparison of the distribution pattern of scores in percentages in quartiles in pre- and post-FCR assessments FCR: flipped classroom.

We further analyzed the distribution of scores in the two assessments in the four quartiles and observed that the students had a significant improvement in their scores after the class time session, with none of the students falling in the lowest (Figure 4 and Table 4).

**Table 4:** Distribution in the percentage of students falling in each quartile based on assessment scores in pre- and post-FCR assessment Chi-square test with two-tailed p-value < 0.0001. FCR: flipped classroom.

Quartiles	Pre-FCR (percentage of students in each quartile)	Post-FCR (percentage of students in each quartile)
0-25	12.0	0.0
25-50	25.0	12.0
50-75	55.0	51.0
75-100	8.0	37.0

Part III: subjective feedback

Students’ Feedback

The feedback of students was taken principally on two aspects: the usefulness of FCR as a teaching module and the utility of individual components of the FCR module. The last part of the feedback was open-ended for any suggestions or comments by the students.

The first part of the feedback questionnaire was further subdivided into three parts: feedback on preparatory learning resources, class time, and overall FCR session (Table 5).

**Table 5:** Students' feedback on the usefulness of flipped classroom

Questions	Number of responses (percentage)					Average Likert scale
	Strongly disagree	Disagree	Neutral	Agree	Strongly agree	
Feedback on preparatory learning resources						
Preparatory material helped me gain an adequate understanding of the topic and enhanced my participation in class time	4 (2.5)	9 (5.7)	28 (7.6)	86 (54.1)	32 (20.1)	3.83
Feedback on class time						
Learning in a group with peers helped effectively in clarifying my queries and improved my conceptual understanding	3 (1.9)	6 (3.8)	15 (9.4)	81 (50.9)	54 (34)	4.11
Overall feedback on flipped classroom (FCR) learning session						
FCR improved my learning efficiency	3 (1.9)	5 (3.1)	23 (14.5)	81 (50.9)	47 (20.9)	4.03
FCR was helpful in motivating me for self-learning	4 (2.5)	1 (0.6)	16 (10.1)	60 (37.7)	78 (49.1)	4.30
FCR helped me apply previous knowledge better than lecture	3 (1.9)	5 (3.2)	18 (11.4)	81 (51.3)	51 (32.3)	4.06
FCR helped integrate physiology with clinical medicine	3 (1.9)	0 (0)	12 (7.5)	77 (48.4)	67 (42.1)	4.28
FCR helped in better concept clarification	2 (3.1)	1 (0.6)	21 (13.2)	80 (50.3)	55 (34.6)	4.16
I prefer FCR over the traditional lecture	7 (4.4)	12 (7.5)	36 (22.6)	49 (30.8)	55 (34.6)	3.83
Overall (average)	2.6%	2.5%	14.4%	44.9%	35.6%	

The second part of the feedback required the students to rate the individual components of the FCR module. All the components' average Likert scale was close to point 4, specifying that they were found to be meritorious (Table 6).

**Table 6:** Students' feedback on the usefulness of individual components of the flipped classroom 1 - If you did not find it useful at all (can do away with the component); strongly disliked it. 2 - Not useful; disliked it. 3 - Neutral about the usefulness. 4 - Useful; liked it. 5 - If you found it extremely useful; strongly liked it. iRAT: individual readiness assurance test; tRAT: team readiness assurance test.

Questions	Number of responses (percentage)					Average response
	1	2	3	4	5	
Preparatory material	4 (2.5)	10 (6.3)	48 (30.2)	79 (49.7)	18 (11.3)	3.60
Assessment	0 (0)	4 (2.5)	39 (24.5)	75 (47.2)	41 (25.8)	3.96
Application questions	0 (0)	5 (3.1)	16 (10.1)	65 (40.9)	73 (45.9)	4.29
iRAT/tRAT	0 (0)	6 (3.8)	29 (18.2)	68 (42.8)	56 (35.2)	4.09

The last part of the feedback form was open-ended and asked for any other suggestions or comments (Table 7).

**Table 7:** A few interesting comments/suggestions of students FCR: flipped classroom.

Comments/suggestions
It was good and directive
More deeper learning than lectures
I really loved the session. Learned a lot, and it was fun discussing everything with my friends. And our teacher discussed it more elaborately in the end. So, I could not find anything more to suggest
Good interactive procedure
This method helped boost my confidence
Increase the frequency of FCR
Difficult and long
First, the topic must be taught in class, then I think reading material will be more effective
Since I had a group of 10 people, I did not get a chance to interact deeply. The groups should be made smaller so that every single individual gets exposed to the discussion and does not feel lost
More than 2 hours can be given for the FCR session

Teachers’ Feedback

Three teachers were actively involved in this FCR exercise, and their feedback was also taken regarding the conduction of this teaching modality. The feedback questions were similarly structured to those of students into three sections: feedback on the utility of FCR, the usefulness of individual components, and lastly, any suggestions or comments (Tables 8-10).

**Table 8:** Teachers’ feedback on the usefulness of the FCR session FCR: flipped classroom; iRAT: individual readiness assurance test; MCQ: multiple-choice question.

Questions	Responses
Feedback on preparatory learning resources	
How much time did you spend on the preparation of pre-class learning resources?	The average time was 9-10 hours insisting on the amount of time required for the development of preparatory material and the laborious work involved in it. Interestingly, even after that, only 74.2% of students found it beneficial, showing the importance of validation. Further improvement in learning resources is required according to student feedback for further sessions
Preparatory material helped the students gain an adequate understanding of the topic and enhanced their participation in the class time	Two strongly agreed while one teacher was neutral, which is in sync with the student responses regarding the preparatory material that most felt it helped while a few thought it could be made better
How much extra effort was required for the preparation of pre-class material and planning for class time?	All three in unison believed that FCR conduction required more effort than the traditional lecture
Feedback on assessment and iRAT	
How much time did you spend on the preparation of the assessment?	The average time was 6-8 hours, again emphasizing the commitment of time required for the development of an effective assessment for the FCR session
How much time did you spend on the preparation of iRAT?	iRAT was based on MCQ-type questions; only the average time required for the same was less than the assessment (2-4 hours) but as this had to be carefully planned to assess concepts explained in pre-class material, it has a certain degree of complexity as well; they involved more time as compared to traditional MCQ preparation
How much extra effort was required for the preparation of the assessment?	Two felt the effort required to prepare an assessment for the FCR session was more than a traditional assessment (as it should not only test the information provided in pre-class material but also allow the student to apply higher learning) while one considered it the same as traditional assessment
How much extra effort was required for the preparation of iRAT?	This answer fetched mixed responses, where one felt not much effort was required for MCQ preparation, one felt it was the same as a traditional lecture, while one felt the workload was more
Feedback on class time	
Learning in a group with peers helped the students in effectively clarifying their queries and improved their conceptual understanding	All three agreed that peer learning aided them in better understanding (two agree and one strongly agree)
How much time is taken for preparation of class time?	The teachers spend a time of around a week to even 15 days preparing for the class time, showing the amount of dedication required to successfully conduct an effective session of FCR
How much extra effort was taken for the preparation of class time?	Two teachers felt there was an extra effort for conduction of class time (to ensure class dynamics, active participation, discipline, etc.) while one felt this is an active learning modality and it was a lesser workload for the teacher
How difficult it was to ensure discipline during class time?	Two felt it was the same as a traditional lecture while one felt it was more difficult (because at times the group start chatting to each other)
How difficult it was to ensure active student involvement during class time?	Two felt that it is an active learning methodology so it was not difficult while one felt it is similar to traditional lecture
Overall feedback on the FCR learning session	
Flipped classroom improved the students learning efficiency	All three agreed that FCR helped in improving the students learning efficiency (two agree and one strongly agree)
Flipped classroom was helpful in motivating students for self-learning	All three agreed that FCR helped in improving the students learning efficiency (two agree and one strongly agree)
Flipped classroom helped apply their previous knowledge better than the lecture	All three agreed that FCR helped in improving the students learning efficiency (two agree and one strongly agree)
Flipped classroom helped students to integrate physiology with clinical medicine	All three agreed that FCR helped in improving the students learning efficiency (two agree and one strongly agree)
Flipped classroom helped students in better concept clarification	All three agreed that FCR helped in improving the students learning efficiency (two agree and one strongly agree)
I prefer flipped classroom over the traditional lecture	Interestingly, two teachers were neutral on this and one agreed. This also goes in line with the students' response to this question where only 65.8% felt it can replace the traditional lecture

**Table 9:** Teachers' feedback on the usefulness of individual components of the FCR module FCR: flipped classroom; iRAT: individual readiness assurance test; tRAT: team readiness assurance test.

Questions	Response
Preparatory material	2 selected Likert scale 5 and 1 Likert scale 4
Assessment	All 3 selected Likert scale 4
Application questions	2 selected Likert scale 5 and 1 Likert scale 4
iRAT/tRAT	2 selected Likert scale 4 and 1 Likert scale 2

**Table 10:** A few interesting comments/suggestions of the teachers

Comments/suggestions
Logistics should be easily available. The topic has to be selected very carefully so that the optimum amount of pre-class material can be shared and there is not too much to cover in class time.

## Discussion

**Figure 5:**
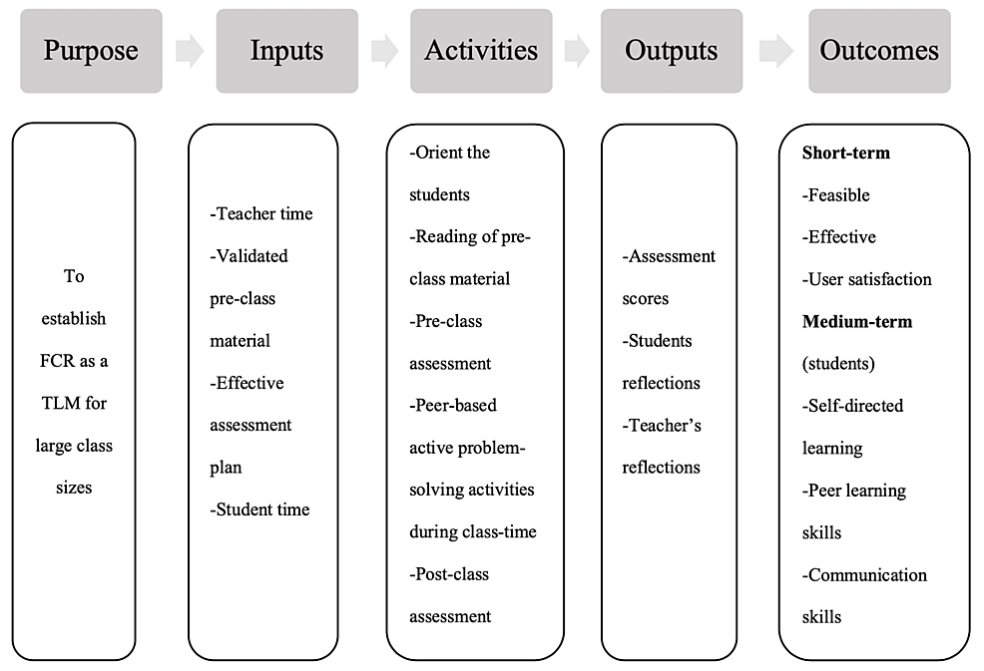
The logic model for planning, implementation, and evaluation of flipped classroom module as a teaching-learning method for large classroom set-up FCR: flipped classroom; TLM: teaching-learning methodology.

This study was designed to establish FCR as a feasible and effective active student-centred teaching-learning methodology in large classroom settings. Figure 5 discusses the logic model of this study.

The structured team-based approach during the in-class session and obtaining student and teacher feedback with a mixed-method approach can be considered major highlights of this study. This study involved a multi-step process listed as follows: (a) planning and preparation for conduction of FCR; (b) conduction of pre-class and in-class sessions; (c) collection of feedback from students and teachers; (d) assessing perspectives of students and teachers to conclusively decipher the benefits and feasibility of the FCR model.

Planning and preparation for conduction of FCR

The planning section involved brainstorming sessions amongst the involved faculty members to decide the topic to be flipped. The topic of "hemostasis" was chosen for FCR on its immense clinical relevance, the scope for active discussion and team learning, and its importance from an examination point of view. The pre-class study material was chosen as a video, the most typical and preferred format reported in the literature [19,20]. The planning was followed by the preparation of pertinent pre- and post-class assessments in line with the specific learning objectives of FCR. The entire timeline of the in-class activity was chalked out, and finally, the challenging activity of construction of application exercises to promote active learning and critical thinking amongst the students was performed. This initial but one of the most important planning and preparation steps required considerable time. This is corroborated by the subjective feedback wherein all three teachers found that preparation for FCR was more time-consuming and required extra effort than traditional lectures. This finding is also in concurrence with earlier studies [17]. After the preparation of the video lecture, it was validated by the Delphi method by three subject experts (the involved teachers in FCR). Based on their feedback, it was felt that a single video was too long, which might hinder the students viewing of the same. Hence, two short videos supplemented with short written texts were finally shared as the pre-class reading material for FCR. This improvisation in the original plan underscores the importance of selecting appropriate topics for FCR sessions. One faculty in subjective response had also suggested that the right topic selection is of utmost importance. The topic should be such that it involves higher-order critical thinking, is highly relevant to the subject, as well as short and crisp so that it can be covered through short video lectures as per the suggestions for effective study material for FCR in previous literature [20].

This created resource material was shared seven days before the actual class to let the students read the study material at their own pace, thus ensuring fulfillment of one of the primary requirements for an FCR session.

Pre-class assessment

The students were subjected to the pre-FCR assessment one day before the in-class session. The average score of students was 54.5%, which probably indicates that students read the study material but still required further clarification about the topic. Based on this pre-class assessment, 12 teams with uniform distribution of low and high-percentile students were made, and the students were instructed to sit in the designated team-wise manner during class time.

Class time

The in-class session was started with iRAT, in which the class responded fairly well. iRAT is for individual accountability for pre-class preparation and testing students' understanding and knowledge of basic concepts needed for the further session, so it seems most students tried to go through the preparatory material provided. The students then solved the same questions as a team. Scores in tRAT were higher than iRAT, although not statistically significant. This definitely underscores the importance of team-based learning, especially in adult learners. In a study comparing traditional learning with flipped learning in a large classroom, they showed during class time of FCR a significant increase in correct answers after discussion with classmates [18].

This was followed by the summarization of important concepts and clarification of doubts of students on the topic. The 12 teams were randomly assigned seven critical order application exercises. Each team subsequently discussed them with other classmates. This session was highly interactive, with different teams giving their inputs and viewpoints regarding each application exercise and narrowing it down to the best-fitting answers.

Finally, the post-class assessment and subjective feedback of students and teachers were taken.

Post-class assessment

As contemplated, the average class score increased, suggesting a significant improvement in their performance. However, there existed a difference in the number of students who attempted the pre-and post-assessment. To address this issue, the pre- and post-test scores of 113 students who had attempted both were compared. This, too, revealed a significant increase in their median scores. Along similar lines, Aristotle et al. found a significant increase in performance in 10 MCQs in comparison to pre- and post-FCR assessments in large class sizes [16]. MCQ as an assessment format suffers from many intrinsic constraints, e.g. results may be biased by reading ability or test-wiseness, tend to focus on low-level learning objectives, and cannot effectively measure students’ ability to organize and express ideas. Thus, the assessment format in our study included a blend of questions, i.e., pictorial fill-in-the-blanks, MCQs, and short answer questions, so that we could assess all the learning domains. Further, the change in performance in terms of delta change (difference between pre and post) scores was analyzed after dividing them into quartiles based on their initial performance. We may argue that any TLM would be expected to improve performance, but strikingly, it was found that low performers had the highest delta change score of 8.64, while for the high performers, the delta change found was only 0.45. It is clearly evident from the aforementioned analysis that poor performers seem to have benefitted the most after the in-class FCR session. This is further supported by the finding that 12% of students had fallen in the lowest quartile in the pre-test, whereas none fell into the lowest quartile in the post-test. In concordance, Herrero et al. compared lectures with FCR in a large class and concluded there was significant improvement with FCR only in students who scored below the median [15]. However, this study had not compared the pre- and post-FCR improvement, and also the comparison of lecture and FCR was done on two separate groups of students, thus increasing the potential biases. This is one of the most predominant outcomes of the present study as it further corroborates FCR as a teaching module to actively engage the students, especially the low performers, and ensure deeper learning. Thus, FCR emerges to be an effective TLM for undergraduate medical students as the Cohen's D effect size is high (which signifies its significant impact) as well as it was found to be highly beneficial even for low performers.

Subjective feedback

Most of the students had spent about two to three hours reading the pre-class material. About 74% of students felt that the preparatory material was beneficial for them, suggesting that the pre-class material was probably useful but had further scope for improvement. These results are akin to a previous study conducted for FCR in a large classroom [16]. About 85% of students enjoyed learning and discussing with their peers during class time, further emphasizing the importance of team-based learning. This is a proven advantage of FCR, which has been reiterated adequately in preceding studies on FCR in large groups [16,18]. In contrast, a study by Fakhoury et al. found only 46.9% of students agreeing that the FCR model helped them in working in a team [17]. This disparity could be easily explained on the basis of subject/topic chosen, population, and study design differences. The overall feedback on the FCR session was quite encouraging. Maximum students felt that the session aided and motivated their learning and helped them to apply prior knowledge. This was in concordance with the findings of Aristotle et al. [16]. Of the students, 92% felt that the inclusion of clinical vignettes during the session was very useful and helped integrate basic physiology with clinical applications. Students also felt that FCR was more efficient in clarification of concepts, in line with the existing literature on FCR in a large class [13,16,17]. However, in response to their preference for FCR over traditional lectures, only 65% of students responded positively, while Aristotle et al. reported 82% of students’ preference for FCR over lectures [16]. The difference in the two studies could be attributable to multiple reasons, e.g. topic of conduction, differences in student population, and the quality of study material provided (which in their study was liked by 92% vis-à-vis in our study 74.2%). But in the open-ended question section, few students reported the module to be long. Herrero et al. found that some students disapproved of it as it was more time-consuming and required more effort of pre-reading [15]. Therefore, this result possibly reflects the reluctance of students to step out of their comfort zone of traditional lectures, which require minimal participation from students.

Furthermore, we have also analyzed the usefulness of individual components so as to decipher their usefulness for the conduction of an effective FCR model. This important aspect has not been addressed in any previous existing literature. Although, Fakhoury et al. tried to gauge the usefulness of instructional tools used in FCR [17]. All the individual components were ranked favorably on the Likert scale. Preparatory material was liked by 61%, which is similar to the results of Fakhoury et al. [17]. While the assessment was liked by 73% of students. However, application exercises were the highlight of our class time and were highly appreciated by the majority (86.8%). This is in contrast to Fakhoury et al. where class time was liked by only 50% of students, further providing us optimistic feedback regarding the efficacy of application exercises. iRAT and tRAT were liked by 78% of students [17]. As expected, the components that involved active team-based participation were preferred over those which required individual effort.

Teachers form the cardinal link for the success of any TLM. Yet, strikingly, none of the studies has actually assessed their feedback regarding the conduction of FCR in large class sizes, with the exception of one study by Fakhoury et al., where he mentions the perspective of a single instructor in a few lines [17]. In the present study, feedback was taken from the teachers involved in the preparation and conduction of FCR. Teachers in our study opined that since only 72% of students found the preparatory material beneficial, more improvement and validation of the material were called for.

In the feedback on class time, all teachers felt that peer learning was valuable. The preparation of class time material took about one to two weeks and showed the importance of a dedicated team in conducting a successful FCR. Maintenance of discipline as well as active student participation was somewhat difficult. On the overall feedback on the FCR learning session, two teachers were neutral, while one definitely appreciated the benefits. This goes in line with the student response where only 65% of students felt that FCR could replace traditional lectures. This further emphasizes the fact that a blend of teaching tools is always welcome.

FCR as a TLM was found feasible (could be successfully conducted), effective (post-scores improved, especially in low performers), and the users seemed mostly satisfied, which highlights the use of FCR in a large classroom set-up. This study, for the first time, establishes the FCR model and its overall effectiveness as TLM for undergraduate medical students in large class sizes with both quantitative (objective assessment scores) and qualitative criteria (subjective feedback from both students and teachers). This study emphasizes that the FCR model can be successfully utilized as TLM for undergraduate medical students even in settings with large class sizes (more than 100 students). This hypothesis is supported with the aid of both quantitative (objective assessment scores) and qualitative criteria (subjective feedback from both students and teachers).

In addition to adding to the literature available on FCR in large classroom settings, the strength of this study is the holistic approach adopted by the authors. All major challenges of FCR were addressed; first, the creation of validated pre-class study material; second, the well-planned team-based scheme for optimum usage of class time that ensured active participation of all students and avoided chaos and indiscipline during class; third, quantitative assessment of the efficacy of FCR as a TLM; and last, the qualitative feedback from both stakeholders, i.e., students and teachers. This approach completed the entire cycle of evaluation of any TLM.

## Conclusions

The present study suggests objective improvement in scores of students after successful conduction of FCR in a large classroom (especially low scorers, further building on the putative advantage of FCR over lectures). We also found overall positive subjective feedback from both students and teachers. FCR is an effective active TLM for large classroom settings.

## References

[1] Ramnanan CJ, Pound LD (2017). Advances in medical education and practice: student perceptions of the flipped classroom. Adv Med Educ Pract.

[2] Prober CG, Heath C (2012). Lecture halls without lectures — a proposal for medical education. N Engl J Med.

[3] Moffett J (2015). Twelve tips for "flipping" the classroom. Med Teach.

[4] Sharma N, Lau CS, Doherty I, Harbutt D (2015). How we flipped the medical classroom. Med Teach.

[5] Lage MJ, Platt GJ, Treglia M (2000). Inverting the classroom: a gateway to creating an inclusive learning environment. J Econ Educ.

[6] Mazur E (2009). Farewell, lecture?. Science.

[7] Pickering JD, Roberts DJ (2018). Flipped classroom or an active lecture?. Clin Anat.

[8] van den Berg EE, Bracey A, van Driel AP, Geijsel FE, Manders S (2016). Modular continuing professional development for emergency physicians - the MNSHA masterclass programme. Eur J Emerg Med.

[9] Veeramani R, Madhugiri VS, Chand P (2015). Perception of MBBS students to "flipped class room" approach in neuroanatomy module. Anat Cell Biol.

[10] Adams NE (2015). Bloom's taxonomy of cognitive learning objectives. J Med Libr Assoc.

[11] Dewey J (1896). The reflex arc concept in psychology. Psychol Rev.

[12] Lucardie AT, Busari JO (2017). The flipped classroom as a pedagogical tool for leadership development in postgraduate medical education. Educ Sci.

[13] Sajid MR, Laheji AF, Abothenain F, Salam Y, AlJayar D, Obeidat A (2016). Can blended learning and the flipped classroom improve student learning and satisfaction in Saudi Arabia?. Int J Med Educ.

[14] Morton DA, Colbert-Getz JM (2017). Measuring the impact of the flipped anatomy classroom: the importance of categorizing an assessment by Bloom's taxonomy. Anat Sci Educ.

[15] Herrero JI, Quiroga J (2020). Flipped classroom improves results in pathophysiology learning: results of a nonrandomized controlled study. Adv Physiol Educ.

[16] Aristotle S, Subramanian S, Jayakumar S (2021). Effectiveness of flipped classroom model in teaching histology for first-year MBBS students based on competency-based blended learning: an interventional study. J Educ Health Promot.

[17] Fakhoury HM, A Fatoum H, Aldeiry MA (2021). Flipping a biochemistry class within a medical curriculum: impacts on perception, engagement, and attainment. Biochem Mol Biol Educ.

[18] Hernández-Guerra M, Quintero E, Morales-Arráez DE (2021). Comparison of flipped learning and traditional lecture method for teaching digestive system diseases in undergraduate medicine: a prospective non-randomized controlled trial. Med Teach.

[19] Fatima SS, Arain FM, Enam SA (2017). Flipped classroom instructional approach in undergraduate medical education. Pak J Med Sci.

[20] Hew KF, Lo CK (2018). Flipped classroom improves student learning in health professions education: a meta-analysis. BMC Med Educ.

